# Management of *Candida guilliermondii* joint infection in a dog

**DOI:** 10.1186/s13028-016-0227-2

**Published:** 2016-07-08

**Authors:** Antonello Bufalari, Chiara Maggio, Giulia Moretti, Alberto Crovace, Valentina Stefanetti, Reinhard Konrad Straubinger, Fabrizio Passamonti

**Affiliations:** 1 Department of Veterinary Medicine, University of Perugia, Via S. Costanzo n.4, 06126 Perugia, PG Italy; 2 Clinica Veterinaria Orvieto, Strada del Piano 9, 05018 Orvieto Scalo, TR Italy; 3 Institute of Infectious Diseases and Zoonoses, Faculty of Veterinary Medicine, Ludwig Maximilians Universität Munich, Munich, Germany

**Keywords:** Dog, Joint infectious, Fungal synovitis, Fungal arthritis, *Candida guilliermondii*

## Abstract

**Background:**

*Candida* spp. are dimorphic fungi in the family Cryptococcaceae. Infections with *Candida* spp. are usually rare conditions in dogs, but immunocompromised patients have a higher risk for developing invasive candidal infections.

**Case presentation:**

A 5-year-old male Boxer, positive to *Leishmania infantum*, was referred to the Veterinary Teaching Hospital of the Department of Veterinary Medicine, University of Perugia, Italy for examination of a non-weight bearing left hind limb lameness of a duration of at least 3 months. During this period, treatment involved systemic anti-inflammatory medications and intra-articular corticosteroid administration. On presentation, clinical examination and radiographic findings were suggestive of cranial cruciate ligament deficiency. To support this diagnosis a stifle arthroscopy was performed: it confirmed a partial rupture of cranial cruciate ligament. Samples culture of synovial fluid and membrane was routinely collected as well, and revealed *Candida guilliermondii* joint infection. Treatment for the *C. guilliermondii* joint infection involved systemic anti-fungal therapy, joint lavage and intra-articular administration of antifungal drugs. Lameness improved markedly during this treatment, but lameness did not resolve completely, probably due to cranial cruciate ligament deficiency. Tibial tuberosity advancement (TTA) was chosen in order to treat stifle instability and was performed 4 weeks following cessation of treatment of the *C. guilliermondii* joint infection. Six month after TTA the dog showed a completely recovery with no lameness.

**Conclusions:**

To the authors’ knowledge, this is the first case of *Candida* spp. joint infection reported in dogs. The cause of the progression of the joint *C. guilliermondii* infection remains unclear but it may be associated with leishmaniasis or intra-articular corticosteroid injections. Treatment with systemic and intra-articular anti-fungal therapies was successful. In the evaluation of hind limb lameness in a chronically immunocompromised dog, it would be advisable to consider also an intra-articular *Candida* spp. infection.

## Background

*Candida* species are the most frequently cultured fungi in healthy dogs. They are ubiquitous organisms existing almost exclusively as commensal organisms, which rarely become pathogenic [1]. *Candida* spp. can be isolated in animals from skin and oral, gastrointestinal, upper respiratory, and genital mucosae. In humans, contamination resulting in infection has been reported following iatrogenic compromise of dermal integrity [1]. In human medicine *Candida* spp. synovitis/arthritis has been associated with intra-articular administration of steroids in order to treat different kind of pathologies such as osteoarthritis and rheumatoid arthritis [2]. The most used intra-articular treatments are methylprednisolone acetate (40–80 mg), triamcinolone hexacetonide (20–40 mg) and triamcinolone acetonide (40 mg); the duration of treatments depends on the type of disease [2]. Intra-articular glucocorticoids may impair local immune defences. The importance of aseptic technique during intra-articular administration of glucocorticoids, in order to decrease the occurrence of secondary infection, has been documented [2, 3]. Whilst reports of bacterial synovitis/arthritis in dogs are non-uncommon, reports on Candida-associated arthritis have apparently not been published yet, although fungal joint infections have been reported in horses [4–8]. The purpose of this case report is to document a successful treatment with a combination of joint lavage, systemic and local antifungal drugs of a solitary case of a *Candida guilliermondii* joint infection in a dog.

## Case presentation

A 5-year-old Boxer with a body weight of 30 kg was admitted to the Veterinary Teaching Hospital of Perugia University, Italy for evaluation of a left hind limb lameness due to a suspected left cranial cruciate deficiency. Lameness had been present for at least 3 months. During that period, treatment was carried out by other veterinarians and had involved systemic anti-inflammatory medication for 2 weeks and intra-articular glucocorticoid administration (drugs and dosages not assessable). Clinical examination revealed reluctance to walk, grade 5/5 lameness based on the Quinn scale [9] and moderate left hind limb muscle atrophy presumed to be secondary to disuse. At rest, the dog demonstrated toe-touching or non-weight bearing left hind limb lameness. Examination revealed a moderate thickening over the medial aspect of the proximal tibia consistent with medial buttress [10]. Manipulations of the joint elicited severe resentment. The cranial drawer and tibial thrust tests were inconclusive. Neurological examination was unremarkable. Examination of the lumbar and lumbosacral spine and hips did not elicit any reaction. After sedation with dexmetedomidine^1^ (4 μg/kg intravenously [IV]), a radiographs of the pelvis and the left hind limb were taken and revealed peri-articular new bone formation in the medial part of the stifle consistent with moderate osteoarthritis and increased synovial fluid volume (effusion). Haematology revealed moderate anaemia (red blood count [RBC]4.87 × 10^6^ cells/µl, Hematocrit [Hct] 33.8 %, complete blood count [cBC] 12.4 × 10^3^ cells/µl) and high level of total proteins (9.3 g/dl), while biochemical parameters were within normal range. A possible explanation for the elevated total proteins and anaemia was leishmaniasis and testing subsequently confirmed *Leishmania infantum* specific antibodies (1/160 Immunofluorescence antibody test [IFAT]). Left stifle arthroscopy was performed. Samples of synovial fluid and synovial membrane and villi were collected and submitted for culture and sensitivity testing. Before premedication with acepromazine^2^ (10 μg/kg IV) and methadone^3^ (0.2 mg/kg IV), carprofen^4^ (4 mg/kg, IV) was administered preemptively [11]. General anaesthesia was induced with propofol^5^ (3 mg/kg IV), and maintained after endotracheal intubation with a mixture of isoflurane^6^ and oxygen (50–100 ml/kg/min) via a circle breathing circuit with spontaneous ventilation. The left hind limb was prepared for aseptic surgery: trichotomy and aseptic disinfection were performed with a wide margin around the left stifle extending from the proximal third of the femur to the distal third of the tibia. Prior to arthroscopy samples of synovial fluid were collected by needle arthrocentesis after a small (8 mm) stab incision. The arthroscopic examination was performed using an arthroscope^7^ connected to a video camera and image-recording device.^8^ The arthroscope was introduced at the level of Gerdy’s tubercle, 2 mm lateral to the patellar tendon. The joint cavity was carefully explored and three samples of synovial membrane were gathered with a 2 mm biopsy forceps. Menisci were examined by visualization and probing as recommended [12]. A small longitudinal tear was identified in the cranial horn of the medial meniscus, more precisely in the cranial tibial ligament of the medial meniscus nearby the intermeniscal transverse ligament. In addition, the craniomedial band of cranial cruciate ligament was ruptured, while the caudolateral band appeared grossly intact. The joint was flushed continuously during joint exploration with sterile saline using a proximal medial parapatellar outflow cannula. At the end of surgery, the arthroscope and surgical instruments were removed and the joint was flushed copiously with lactated Ringer’s solution [13]. After recovery from anaesthesia, to provide postoperative analgesia, buprenorphine^9^ (0.015 mg/kg subcutaneously [SC]) was administered three times a day [TID] for 1 day while carprofen (see footnote 4) (2 mg/kg SC) was given once a day for 10 days. Laboratory findings of the synovial fluid are reported in Table 1.

**Table 1:** Laboratory findings of the synovial fluid

Colour	Appearance	Quantity (ml)	Protein (g/dl)	WBC (cells/µl)	RBC (cells/µl)	Glucose (mg/dl)
Reddish	Cloudy	12	3.5	35,000	250	<10

*WBC* white blood count, *RBC* red blood count

**Fig. 1:**
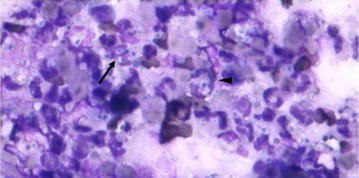
Cytological appearance of the synovial fluid. 4–6 µm wide yeasts (*arrow*) and some neutrophils containing yeasts (*arrowheads*) were observed with May-Grünwald-Giemsa

Smears from synovial fluid were stained with May Gruenwald-Giemsa and examined under a light microscope. The sample showed a blue proteinaceous background and a mixed inflammatory cell population, with some yeasts as shown in Fig. 1. Synovial fluid and small fragments of the capsule were pre-enriched on 5 ml of triptic soy broth (TSB) medium and incubated at 37 °C. After 48 h, 100 µl of each broth cultures was subcultured for aerobic bacteria and fungi on blood agar, MacConkey agar and Sabouraud dextrose agar at 37 °C in 5–10 % CO_2_. No bacterial growth was observed but a massive growth of yeast with the morphology of *Candida* spp. was isolated after 24 h of incubation from both synovial fluid and the fragments of synovial tissue. The identification of *Candida* spp. was based on phenotypic feature such as a description of the macro- and micromorphology and through fermentation of carbohydrates and auxanographic using a commercial yeast identification (Api 20 C Aux Biomerieux^10^). Colonies grown on Sabouraud dextrose agar were subjected to polymerase chain reaction (PCR) amplification and sequencing analysis in order to determine the *Candida* species. One fungal colony was transferred in a 1.5 ml tube containing 200 µl of distillated water. DNA was extracted using the QIAamp DNA mini kit^11^ in accordance to manufacturer’s instructions and the D1/D2 region of the 26S rRNA gene was amplified by PCR assay, as previously described [14]. PCR positive reactions were purified using Wizard SV Gel and PCR Clean-up System (Promega, Madison, WI, U.S.A.) in accordance with the manufacturer’s recommended protocol and subjected to direct sequencing. Consensus sequences were created with the BioEdit Sequence Alignment Editor Software v 7.0.9.0 and then searched against the Genbank database. The isolated *Candida* sp. was thereby identified as *C. guilliermondii.*

Before treatment was initiated with antifungal drugs, sampling of synovial material was repeated with the same aseptic procedure and growth of *C. guilliermondii* was confirmed. Culturing of blood and urine samples did not produce *Candida* colonies and consequently systemic candidiasis was rejected. Antifungal therapy was started with fluconazole^12^ (5 mg/kg per oral [PO] for 10 days; once a day). The intra-articular treatment was performed under general anaesthesia: after premedication with dexmedetomidine (see footnote 1) (4 μg/kg Intramuscular [IM]) and methadone (see footnote 3) (0.2 mg/kg IM), general anaesthesia was induced with propofol (see footnote 5) (3 mg/kg IV), and maintained as previous described. The left hind limb was then prepared for aseptic procedure and copious joint lavage (30 ml/kg of lactated Ringer’s solution) and intra-articular administration of 10 ml (0.7 mg/kg) of 0.2 % fluconazole^13^ solution were performed on alternate days for three times. Synovial fluid analysis after 9 days of treatment, failed to culture *C. guilliermondii*. Two weeks following initiation of intra-articular treatment, clinical examination revealed significant improvement in function with 1/5 left hind limb lameness (lameness barely detectable) and no resentment to manipulation of the left stifle. Radiography of the stifle revealed a similar grade of osteoarthritis but an evident decrease of synovial volume. Systemic treatment for leishmaniasis was instituted. New samples of synovial fluid from the left stifle were collected during TTA 1 month later and sent to the same diagnostic laboratory involved for previous testing. These samples were negative for bacterial and fungal growth (serial clinical procedures and treatments carried out are listed in Table 2). Six month after TTA surgery the referring veterinarian reported that the dog showed a clinical improvement with no lameness (0/5).

**Table 2:** Serial clinical procedures and treatments

Day	Clinical procedures	Laboratory procedures	Farmacological treatments
0^a^	Orthopaedic examination, radiographic study, first arthroscopy to collect synovial fluid and synovia (membrane and villi) and to explore stifle joint	Blood and synovial analysis, bacterial culture and sensitivity testing	Perioperative analgesic treatment: carprofen 4 mg/kg IV SID and buprenorphine 0.015 mg/kg IM TID
1		Cytological examination of synovial revealed yeasts. Serological test positive for *Leishmania infantum*	Analgesic treatment: carprofen 2 mg/kg SC SID and buprenorphine 0.015 mg/kg IM TID
2		Positive culture for *Candida* spp. (post enrichment)	Analgesic treatment: carprofen 2 mg/kg SC SID
3	Second arthroscopy to obtain new samples and carry out joint lavage	New bacterial culture and sensitivity testing of synovial fluid and synovia (membrane and villi)	Perioperative analgesic treatment: carprofen 2 mg/kg IV SID and buprenorphine 0.015 mg/kg IM TID
4			Analgesic treatment: carprofen 2 mg/kg SC SID and buprenorphine 0.015 mg/kg IM TID
5	Starting antifungal treatment: joint lavage + intra-articular fluconazole (0.7 mg/kg)	Confirmation of positivity for *Candida* *guilliermondii*	Antifungal and analgesic treatment: fluconazole 5 mg/kg PO SID, carprofen 2 mg/kg SC SID, respectively
7	Joint lavage + intra-articular fluconazole (0.7 mg/kg)		Antifungal and analgesic treatment: fluconazole 5 mg/kg PO SID, carprofen 2 mg/kg SC SID, respectively
9	Joint lavage + intra-articular fluconazole (0.7 mg/kg)	Bacteriological culture of synovial fluid	Antifungal and analgesic treatment: fluconazole 5 mg/kg PO SID, carprofen 2 mg/kg SC SID, respectively
10		Antifungal and analgesic treatment: fluconazole 5 mg/kg PO SID, carprofen 2 mg/kg SC SID, respectively	
11	Returned to the care of the primary practitioner	Negative to *Candida* spp.	
19	Follow up: improvement of lameness (1/5)		Starting *Leishmania* treatment (by the practitioner)
49	TTA surgery (by the practitioner).	New sample of synovial fluid for bacteriological culture during TTA surgery	
51		Confirm negativity to *Candida* spp.	

^a^Lameness had been apparent for approximately 3 months prior to the day of referral (denoted day 0)

*Candida* spp. are opportunistic pathogens often causing severe systemic infections in immunocompromised patients [15, 16]. In humans, *Candida* spp. induced-arthritis is a rare disease and most of the cases have been also associated with immunosuppressive conditions such as cancer, AIDS, organ transplantation, chronic renal failure, steroid use, and heroin abuse [17–19]. Although virtually any joint can be affected, large, weight-bearing joints such as the knees are most commonly involved [15]. *Candida guillermondii* is widely distributed in nature, frequently isolated from the environment and it is a part of saprophyte human and animal microflora on the skin and mucosal surfaces. However, it is considered to be an uncommon causative agent of disease [20]. Reports of *Candida* spp. infections in dogs are rare as well. *Candida guilliermondii* was isolated from cutaneous candidiasis in a dog [21]. Systemic candidiasis has been reported in dogs causing signs of acute onset with pyrexia and septic shock [22, 23, 24]. Localized candidiasis is often asymptomatic [17], although in chronically immunosuppressed dogs with involvement of the skin, nail beds, urinary tract, ears, and gastrointestinal tract have been described [1, 25]. A single report describes an intra-articular fungal (*Blastomyces* spp.) infection in the carpus of a Labrador retriever, which was treated successfully with itraconazole PO [26]. To the authors’ knowledge this is the first report of a *C. guilliermondii* joint infection in a dog. *Candida* spp. infections have been associated with immunosuppression in dogs and humans [15]. Four mechanisms may have contributed to the development of *C. guilliermondii* infection in the present case: leishmaniasis, intra-articular steroids [27], repeated needle puncture compromising the skin barrier [4, 7], and lymphoplasmacytic synovitis observed especially in Boxer dogs [28]. Although minimally invasive techniques such as joint puncture are not an important risk factor for developing infective arthritis, in our opinion joint injections pose a potential risk for microbiological joint contamination that may evolve in infection especially in attenuated immune response dogs. Intra-articular injections of glucocorticoids are widely used in arthritic human patients [29, 30], despite some evidence that intra-articular steroids do not influence the expression of some important mediators of inflammation and cartilage destruction [31, 32]. It has been reported that bacterial DNA is frequently found in canine synovial tissue from cranial cruciate ligament rupture joints, possibly as a result of altered immune defences, which may relate to lymphoplasmacytic inflammation [28]. In the current case, leishmaniasis may have contributed to destabilize the dog’s immune system [33] by promoting the rooting of the *C. guilliermondii*. Moreover, intra-articular glucocorticoids may also have induced a status of local immune suppression that may have created a favourable environment for *C. guillermondii*. In the authors’ opinion both previously performed intra-articular treatments and the instability due to the cruciate deficiency, maybe have had major roles among the predisposing factors for developing joint fungal infections. In our opinion the elimination of *C. gulliermondii* was indicated prior to treatment of the leishmaniasis due to the possible side effects and toxicity of drugs resulting from the combined treatment. The therapy of leishmaniasis was appropriate prior to surgery to stabilize the stifle in order to reduce the complications that may arise by the oxidative stress caused by the surgical procedure in an immunocompromised patient. Optimal treatment of *C. guilliermondii* arthritis has not been established [17]. Antifungal susceptibility testing is usually unstandardized and unreliable [34], so antifungal agents that are effective against a broad spectrum of medically important *Candida* spp. are recommended. In one recent report, a woman with *Candida* spp. infection of a joint was treated with joint debridement and antifungal medications, such as amphotericin B (0.5–1 mg/kg/day IV) and fluconazole (400 mg/day PO) [17]. Amphotericin was given IV for 3 weeks and PO fluconazole was prescribed for 6 months. Amphotericin B is a polyene antifungal commonly used for overwhelming systemic mycoses and leishmaniosis [1]. Fluconazole is an antifungal drug with higher affinity for fungal enzymes in comparison to ketoconazole or itraconazole. Its bioavailability is elevated after IV or PO administration [1]. A long-term survey of *Candida* spp. infections demonstrated that fluconazole had fewer side effects and a superior antifungal effect than amphotericin B [35]. Treatment of a *Candida* spp. joint infection using fluconazole as a sole agent has been reported in a single human [36]. In human medicine, amphotericin B may have poor absorption after oral administration and is painful when injected IM, potentially nephrotoxic and can exacerbate liver failure. For this reason we chosen fluconazole as treatment of the localized fungal infection. Dosage recommendations vary from 2.5 to 10 mg/kg every 12–24 h for treatment of candidiasis in humans. In this case fluconazole was dosed at 5 mg/kg once a day, which resulted in an effective response [22]. Even in neonatal foals fluconazole (4–5 mg/kg PO) has been used to treat systemic candidiasis and a lower toxicity than amphotericin B was shown [5].

A variety of treatments are available for septic arthritis, but long-term osteoarthritis is inevitable. The treatment options range from systemic drugs, local and regional medications, joint lavage via large bore needles, and arthroscopic lavage, to open arthrotomies for continuous drainage [7, 37]. Although joint lavage has not been found to be effective in slowing the rate of progression of osteoarthritis without sepsis, it is recommended in septic arthritis to remove some of the inflammatory mediators and debris [37]. Large portals allow the removal of purulent debris and the use of a large volume of fluid. Repeated lavage or continuous drainage of the joint may be indicated if clinical signs persist from continued synovitis or production of purulent debris [37]. The decision for choosing the intra-articular route for fluconazole administration was based on the aim to obtain a high concentration of compound in the joint and eliminate the infection more rapidly. This may be debated since in humans a parenteral administration of fluconazole produces substantial synovial fluid levels [38]. In this case we did not observe joint swelling or localized pain nor any other side effects in the following days after administration. As reported in horses [4] intra-articular administration of fluconazole can be actuated without local/systemic complications. *Candida* spp. joint infections need treatment in all cases. Since *Candida* spp. are less potent infectious agents in a healthy immunocompetent patient, the presence of *Candida* spp. infection often indicates a status of immune deficiency. A combined approach for treatment of the *Candida* infection and of the pathology responsible for immunocompromised is necessary.

## Conclusions

To the authors’ knowledge, this is the first documented case of *C. guilliermondii* joint infection in a dog. The objective of this study was to document the successful diagnosis and treatment of fungal infectious arthritis. Since this is the first report on *Candida* spp. joint infection in a dog, no guidelines regarding treatment exist and the decision to administer fluconazole into the joint was made mainly on reports in horses. Fungal arthritis should be considered when encountered by joint infections, particularly in cases with a concomitant immunosuppression. In fact, the diagnosis of septic arthritis with a normally commensal fungal organism should alert the clinician to the possibility of local and/or systemic immunocompromised state. Treatment including repeated joint lavage and administration of systemic and local (intra-articular) fluconazole resulted in eradication of the *C. guilliermondii* infection in this case.

## Abbreviations

IV: intravenous
IM: intramuscular
PO: per os/per oral
SC: subcutaneous
TTA: tibial tuberosity advancement

## Footnotes

1 Dexdomitor^®^: Orion Corporation Orion Pharma, Espoo, Finland.

2 Prequillan®, Fatro Spa, Bologna, Italy.

3 Eptadone®, Molteni Spa, Firenze, Italy.

4 Rimadyl®, Zoetis Inc. New Jersey, Stati Uniti.

5 Proposure®, Merial Italia Spa, Milano, Italy.

6 Isoflo; Esteve Spa, Milano, Italy.

7 2.7 mm, 30° oblique arthroscope, Small Joint Arthroscope: Karl Storz GmbH & Co. KG, Tuttlingen, Germany.

8 Digital Video Camera DC-1300, NeoMed®, Gutach im Breisgau, Germania; Keymat DVD + RW video recorder, Nola, Italy.

9 Temgesic®, RB Pharmaceuticals Limited, Milano, Italy.

10 Api 20 C Aux Biomerieux, Marcy-l´Étoile, France.

11 Qiagen, Hilden, Germany.

12 Diflucan®, Pfizer, New York, USA.

13 Fluconazolo ratiopharm®, Ratiopharm Italia Srl, Assago, Italia.
